# Analysis of the determinants of alcohol consumption and expenditure in Ecuador: an econometric analysis

**DOI:** 10.3389/fpubh.2024.1471578

**Published:** 2025-01-03

**Authors:** Ricardo Yajamín-Villamarín

**Affiliations:** ^1^Pontifical Catholic University of Ecuador, Quito, Ecuador; ^2^University of South Wales, Cardiff, United Kingdom

**Keywords:** alcohol consumption, alcohol expenditure, determinants, probit model, tobit model, Ecuador

## Abstract

**Background:**

This study delves into the determinants of alcohol consumption and expenditure in Ecuador, focusing on personal characteristics, education levels, and regional variations. This study aimed to provide nuanced insights into alcohol-related behaviors within the Ecuadorian population.

**Methods:**

Employing probit and Tobit models, the study ensures a robust analytical framework to assess the factors influencing alcohol consumption and expenditure. Data were collected from the 2014 Survey of Living Conditions, spanning urban and rural areas of Ecuador, guaranteeing a diverse population representation. The study includes individuals from random households, totaling 66,418 individuals over 18 years. Employing various measurements, including the use of a probit model for alcohol consumption and a Tobit model for alcohol expenditure, the study considers key variables such as smoking, gender, age, employment status, and regional location, contributing to a detailed understanding of alcohol-related behaviors.

**Results:**

For female individuals, there is a negative association, decreasing the probability of alcohol consumption by 6.6% (*p*-value: 0.000). Conversely, being a smoker exhibits a positive association, increasing the likelihood by 10.2% (*p*-value: 0.000). Regarding alcohol expenditure, being female is linked to a reduction in spending by $0.885 (*p*-value: 0.000). Being a smoker reveals an increase in spending by $0.914 (*p*-value: 0.000). Regional variations, education level, and employment status play crucial roles in shaping alcohol-related behaviors.

**Conclusion:**

This research provides nuanced insights into the socioeconomic determinants of alcohol-related behaviors in Ecuador. The findings underscore the necessity for targeted public policies, including gender-specific interventions, anti-smoking strategies, and considerations for regional variations.

## Introduction

Public health initiatives on a global scale must pivot toward mitigating the adverse effects of alcohol consumption, a psychoactive substance renowned for its inherent addictive properties (1, 2). Addressing negative drinking habits has the potential to significantly alleviate the detrimental impact on individuals and society at large. This imperative is particularly pronounced in the Latin American region, where persistent challenges demand effective solutions to combat the escalating issues posed by excessive alcohol consumption (3). The Americas, ranking second in alcohol consumption per capita among World Health Organization (WHO) regions, confront a pattern associated with heightened health risks, boasting the second-highest rate of excessive alcohol consumption. In response, several countries within the region have intensified their public health measures, incorporating regulations on alcohol sales, tax increments, and constraints on availability, including enforcing a minimum legal age for consumption and purchase (4).

Beyond health implications, alcohol consumption exerts substantial economic repercussions, spanning healthcare, law enforcement, and lost productivity. The financial burden is particularly notable when resources are diverted from essential needs such as food and education. The economic costs attributed to alcohol encompass three categories: costs incurred by consumers, those borne by immediate social circles, and those absorbed by society as a whole (5). In particular, no society has demonstrated a net economic benefit from alcohol that outweighs the costs incurred due to associated problems. Most of these costs are linked to lost productivity, peaking in the economically productive age range of 15 to 49 years, the same bracket where alcohol-related mortality and disability rates are the highest. Moreover, the harmful use of alcohol results in staggering wage losses in the Americas, potentially amounting to billions of dollars annually (4). Existing studies indicate that more than 1% of GDP is lost in high- and middle-income countries due to the harmful consequences of alcohol consumption (6).

This study aimed to unravel the determinants of alcohol-related behaviors in Ecuador, a country where the constitution recognizes addictions as a public health problem. The government, committed to prevention, control, and rehabilitation programs, underscores the need for a nuanced understanding of alcohol-related behaviors (7). Government policies, particularly those regulating the sale of alcoholic beverages, are integral components of the broader strategy to manage public health and maintain internal security. The Ministry of the Interior, aligned with constitutional mandates, oversees the enforcement of policies related to the sale and consumption of alcoholic beverages, reflecting the commitment of government to maintaining public order (7).

In the Latin American context, Ecuador ranks ninth in alcohol consumption, with individuals consuming 7.2 L of pure alcohol per capita per year, according to 2014 data (2). This underscores the urgency and relevance of studying alcohol-related behaviors within the Ecuadorian context. This study delves into the socioeconomic factors influencing alcohol consumption and expenditure, utilizing comprehensive data from the 2014 Survey of Living Conditions conducted by the National Institute of Statistics and Censuses (8). Employing a probit model for analyzing the probability of alcohol consumption and a Tobit model for assessing expenditure adds methodological rigor to the investigation. Furthermore, the inclusion of pertinent variables, such as smoking habits and engagement in sports, enhances the comprehensiveness of the study.

The determinants of alcohol consumption and expenditure have been extensively studied through a variety of econometric models, which allow for a deeper understanding of how different factors influence these behaviors across countries. These models include Double Hurdle, Tobit, and probit analyses, among others. Such studies emphasize household characteristics, gender, education, income levels, and geographic location as significant predictors of alcohol consumption and spending.

For example, some research has demonstrated that gender plays a key role in alcohol consumption and expenditure. Men are more likely to consume alcohol and spend more on it than women, with education and urban-versus-rural settings also significantly affecting these behaviors (9–11). Similarly, the bivariate probit model used by Alkan et al. found that concurrent use of alcohol and tobacco was influenced by factors such as age, education, and income level, with the probability of alcohol and tobacco use increasing with higher educational attainment (12, 13).

In studies focusing on women, education has been found to significantly increase the likelihood of alcohol consumption. Research by Ünver and Alkan (14) highlighted that younger, educated, single, and employed women in Turkey had a higher propensity to consume alcohol than their counterparts. This is consistent with global trends that show rising alcohol consumption among women due to socioeconomic changes and gender equality movements, although public health risks remain a concern, particularly related to tobacco use and alcohol-related diseases.

Recent research further emphasizes the role of socioeconomic factors. Aksoy et al. (2019) found that male-headed households were more likely to consume alcohol and spend more on it, while higher education and urban residence increased alcohol participation. Additionally, household size negatively correlated with alcohol spending, suggesting that larger families tend to reduce expenditures on non-essential goods like alcohol (15, 16). In Malaysia, the interaction between income, ethnicity, and education affects both the decision to purchase and the amount spent on alcohol. Higher income correlates with increased alcohol consumption across ethnic groups, and urban households spend less on alcohol than rural ones (17). These findings reflect how economic capacity and cultural context shape drinking behaviors.

Ecuador, a developing country, exhibits patterns of alcohol consumption and expenditure similar to those of other emerging economies. Alcohol is considered a luxury good, and socioeconomic factors have a significant impact on consumption patterns (16). As societies grapple with the challenges posed by alcohol consumption, informed and targeted interventions become imperative. This study engages advanced statistical models to explore the intricate interplay of factors influencing alcohol-related behaviors, including gender, education, employment, and regional disparities. By analyzing these determinants, this study aimed to contribute to the literature on alcohol consumption in developing contexts, providing insights that can inform public policy aimed at reducing excessive alcohol use and its associated health and economic consequences.

## Methods

### Data

The dataset used was the Survey of Living Conditions (ECV) 2014, designed and collected by the National Institute of Statistics and Censuses (INEC) in Ecuador. This is a household-level dataset that provides detailed information on alcohol consumption, personal characteristics, and other potential factors associated with alcohol use, such as smoking habits and participation in sports. The survey covers 66,418 individuals aged 18 years or older from urban and rural areas across all 24 provinces of Ecuador. For each household, questionnaires were administered to all individuals, ensuring comprehensive intra-household data collection (8).

These data are statistically representative at the national, regional, and provincial levels. It also allows for analyses at finer levels, such as nine planning zones and four auto-represented cities (Quito, Guayaquil, Cuenca, and Machala). This stratified and two-stage sampling design ensures that the results can be generalized to the entire Ecuadorian population, providing high-quality data to monitor social and economic conditions across regions.

The ECV follows a probabilistic, two-stage sampling design. Primary sampling units (PSUs) were created by clustering census sectors, and these were selected using probability proportional to size (PPS) based on the number of households reported in census cartography. In the second stage, households were randomly selected from within each PSU, giving all households an equal chance of selection through simple random sampling. The sample size was determined by several factors, including the study domains, desired precision, and expected non-response rates. INEC adjusted the sample sizes to account for the design effect and to maintain statistical reliability even in areas with low response rates. Control variables such as unemployment rate, per capita household income, poverty rate, and extreme poverty rate were used to guide sample allocation and ensure consistency across survey cycles (8).

This survey contains a wealth of socio-demographic information, including age, gender, education, employment status, income level, marital status, and geographic location. This comprehensive set of variables provides a robust foundation for analyzing the determinants of alcohol consumption in Ecuador.

### Variables

#### Dependent variables

Table 1 describes the variables, their definition, and the type of variable that each represents in this model. As already discussed, the variable drinker is defined as the participation in the consumption of alcoholic beverages and is of type dummy. While the expenditure variable is defined as the total spending on alcoholic beverages (excluding beer), this variable is continuous. Beer consumption was excluded from the analysis due to differing reporting periods in the ECV: beer data are collected weekly, while other alcoholic beverages are reported monthly, complicating integration.

**Table 1:** Type and definition of dependent and independent variables.

Variables	Definition	Type
Dependent variables		
Drinker	Consumption of alcoholic beverages: 1 if the individual consumed any alcoholic drink (excluding beer) the last month, 0 otherwise	Dummy
Expenditure	Total spending on alcoholic beverages (excluding beer)	Continuous
Independent variables		
Personal characteristics		
Female	1 if female, 0 otherwise	Dummy
Age	Age in years	Continuous
Indigenous	1 if indigenous, 0 otherwise	Dummy
Rural	1 if living in a rural area, 0 otherwise	Dummy
Employed	1 if actively employed, 0 otherwise	Dummy
Cohabiting couple	1 if living with a partner, 0 otherwise	Dummy
Education level		
No education	1 if no education, 0 otherwise	Dummy
Primary school	1 if the primary school was completed, 0 otherwise (reference category)	
High school	1 if high school, 0 otherwise	Dummy
University	1 if the university or higher, 0 otherwise	Dummy
Regions		
Andes	1 if living in the Andes, 0 otherwise (reference category)	Dummy
Coast	1 if living in Coast, 0 otherwise	Dummy
Amazon	1 if living in Amazon, 0 otherwise	Dummy
Galapagos	1 if living in Galapagos, 0 otherwise	Dummy
Control variables		
Smoker	1 if smoke, 0 otherwise	Dummy
Sport	1 if do sport, 0 otherwise	Dummy

#### Independent variables

Explanatory variables were included in line with the evidence from the existing literature. Several studies have found that personal characteristics such as gender, age, ethnicity, living area, employment status, and whether the person lives with their partner or not, are likely to affect individual alcohol behaviors. The education level of each individual can be considered as a proxy variable for the income level (9). Dummy variables were generated with the education level of each person, such as no education, primary education, high school, and university. The university variable considers all types of degrees, including postgraduate or PhD. The regions of Ecuador were considered. There are four regions: Andes, Coast, Amazon, and Galapagos. Each region is different in culture and climate, which can influence alcohol consumption and spending. Finally, two last variables were included, referring to whether the individual had smoked in the last month and whether he had practiced any sport, as shown in Table 1.

### Statistical analysis

#### Alcohol consumption in the last month

The dependent variable was built from the question of whether the individual drank an alcoholic beverage (excluding beer). Therefore, the variable drinker was defined as the participation in alcohol consumption, which took the value of 1 if the individual consumed alcohol and zero otherwise. To analyze the main determinants of the probability of drinking in the month previous to the interview, a probabilistic model of discrete choice was estimated to investigate the characteristics of an individual with a greater or lesser probability of consuming alcohol.

The determinants that affected the individual’s participation to consume or not alcohol were evaluated. Given the dichotomous nature of the dependent variable, a linear probability model would not be the most appropriate option as predicted probabilities are not constrained to lie between 0 and 1. Therefore, a probit model (10) for the binary choice was introduced to account for the dichotomous nature of the drinker variable.

In the probit model, the start point is a latent variable expressed as follows:


$$y^{*}=x^{\prime }\beta +\varepsilon$$

We observe y which is related to $y^{*}$ as follows:


$$y=1\;\text{if}\;y^{*}>0$$

$$y=0\;\text{if}\;y^{*}\leq 0$$

where y is the dependent variable drinker that was studied in this model. It is assumed that this variable can only present two results, namely, y = 0 if the individual did not drink alcohol in the last month and y = 1 if the individual consumed alcohol. This may have some limitations such as normal distribution requirements, but it is the only information available using Ecuador data.

So, the probability that an individual consumes alcohol can be modeled with the following expression:


$$\mathrm{Pr}\;\left(y=1|x\right)=\mathrm{Pr}\;\left(x’\beta +\varepsilon >0\right)=\mathrm{Pr}\;\left(\varepsilon >-x’\beta \right)$$

$$\mathrm{Pr}\;\left(y=1|x\right)=1–\Phi \;\left(-x’\beta \right)=\Phi \;\left(x’\beta \right)$$

The explanatory variables are represented by x’, and the parameter β reflects the impact of x upon the probability of an individual consuming alcohol. Moreover, *ε* is assumed to be an IID standard normal and Φ is the cumulative distribution function for the standard normal distribution (10). To obtain the variation in the probability of observing a positive result succeeding a marginal change in an explanatory variable, it is necessary to obtain the marginal effect which was calculated in the model.

#### Alcohol expenditure in the last month

Similarly, the dependent variable of alcohol expenditure was defined as the expenditure on any type of alcoholic beverage (excluding beer) in the last month. This dependent variable was censored as it contained a large number of zeros. This means that most individuals spent zero dollars on alcohol.

The factors influencing the expenditure of alcoholic beverages were analyzed. A standard regression model such as OLS did not take into account the qualitative difference between non-limit observations and limit observations with several zeros in alcohol expenditure. So, a censored regression model was the most suitable for this study. Taking into account that the nature of the alcohol spending variable was censored by many zeros and the limitations of available data from Ecuador, a Tobit model was used. The Tobit model (18) is a censored regression model and a class of models that contains discrete and continuous parts. The variable alcohol expenditure shows a censoring from below as it is seen in the next expression:


$$y=\{\begin{array}{c} y^{*}\text{if}\;y^{*}>L\\ L\;\text{if}\;y^{*}\leq 0 \end{array}$$

where L is the lower limit; in this case, expenditure equals to zero. The actual value for the dependent variable y is observed if the latent variable $y^{*}$ is above the limit and this limit is observed for the censored observations. Hence, it can be observed the actual expenditure for individuals who bought alcohol and zero for individuals who did not buy alcohol.

The Tobit model is derived as follows:


$$y^{*}=x^{\prime }\beta +e$$

$$y=\{\begin{array}{c} y^{*}\text{if}\;y^{*}>0\\ 0\;\text{if}\;y^{*}\leq 0 \end{array}$$

$$y=\max\left(y^{*},0\right)$$

In this case, the dependent variable is left-censored at zero, meaning the distribution includes many zeros and positive values. Here, y represents expenditure on alcoholic drinks, while $y^{*}$ is the unobserved latent variable that determines the observed value of y. The explanatory variables are represented by $x^{\prime }$, β indicates the effect of x on $y^{*}$, and e is the error term (18).

Many authors used a Tobit model in their research despite its methodological limitations and obtaining impactful findings (18). A Tobit model cannot handle the situation in which participation and spending on alcohol may be separate decisions; this could be influenced by different determinants or by the same variables but in different ways.

Conversely, other studies have used other variants to model censored variables and these models are known as double hurdle models such as the Heckman sample selection model or Cragg model. The main difference between a Tobit model and a double hurdle model is that double hurdle models relax the restrictive assumption of the Tobit model that the discrete decision and the continuous decision are the same. For instance, the Heckman model differs from the Tobit model in that it is possible to observe the process in a two-step or stage decision and thus allow the use of different sets of independent variables in both stages of the estimates. On the other hand, the Tobit model uses a one-step method as it assumes that the independent variables affect the decision to participate and that the level consumed is the same (19).

In Ecuador, no database allows studying the alcohol expenditure variable through a double hurdle model; that is, there is a lack of data in which a set of different variables can be used to investigate the individual’s decision to participate in the consumption of alcoholic beverages and another set of different variables to investigate their spending. Literature is vast at the time of using a model that allows evaluating censored variables such as spending on alcoholic beverages. It is important to take into account the limitations of each model and apply the one that best fits the objectives set and the available data. In this case, the Tobit model, despite its limitations, was the most appropriate for this study. Regression estimations were weighted, using expansion factors to make the results representative of the target population. This approach allows the estimates to be adjusted to accurately reflect the distribution and characteristics of the sample within the analysis context.

## Results

### Descriptive statistics

The data show that 13.22% of respondents are drinkers. The distribution is presented by both gender and region within Ecuador, with a higher prevalence of consumption among men and in the Andes and Costa regions. Additionally, the mean expenditure among drinkers is 7.388 USD, with a standard deviation of 13.986. Table 2 summarizes the descriptive statistics of drinkers distributed by gender and region.

**Table 2:** Descriptive statistics of the drinkers distributed by gender and region.

Variable	Drinker (%)	No drinker (%)	Total
Is the person considered a drinker?	8,782 (13.22%)	57,636 (86.78%)	66,418
Distribution by gender			
Male	7,021 (80%)	25,083 (44%)	32,104
Female	1,761 (20%)	32,553 (56%)	34,314
Distribution by region			
Andes	3,947 (45%)	29,048 (50%)	32,995
Coast	3,288 (37%)	18,927 (33%)	22,215
Amazon	1,384 (16%)	8,661 (15%)	10,045
Galapagos	163 (2%)	1,000 (2%)	1,163
Drinker expenditure			
Mean	$ 7.388		
Standard deviation	13.986		

The results of the probit model examining the determinants of alcohol consumption and expenditure reveal intricate associations across various socio-demographic factors. Tables 3, 4 provide a comprehensive overview of estimated coefficients, standard errors, *p*-values, and 95% confidence intervals, allowing for a detailed understanding of the nuanced influences on alcohol consumption and expenditure.

**Table 3:** Results of probit model for alcohol consumption during the previous month (*n* = 66418).

Independent variables	Marginal effects				
	Estimated coefficient	Standard error	*p*-value	95% Confidence interval	
Personal characteristics					
Female	−0.066***	0.003	0.000	−0.072	−0.061
Age	−0.000	0.000	0.515	−0.000	0.000
Indigenous	−0.006	0.004	0.106	−0.014	0.001
Rural	0.002	0.003	0.506	−0.004	0.007
Employed	0.022***	0.004	0.000	0.016	0.029
Cohabiting couples	−0.005	0.003	0.127	−0.012	0.001
Education level					
No education	0.003	0.006	0.613	−0.009	0.016
High school	0.007*	0.003	0.032	0.001	0.014
University	0.030***	0.005	0.000	0.020	0.041
Regions					
Coast	−0.028***	0.003	0.000	−0.035	−0.022
Amazon	−0.025***	0.003	0.000	−0.031	−0.019
Galapagos	−0.044***	0.007	0.000	−0.057	−0.031
Control variables					
Smoker	0.102***	0.006	0.000	0.090	0.114
Sport	0.015***	0.004	0.000	0.008	0.022

Standard errors are clustered at the household level. ^*^p < 0.05; ^**^p < 0.01; ^***^p < 0.001. Primary education is the reference category for education level. The Andes is the reference category for the region.

**Table 4:** Results of tobit model for alcohol expenditure during the previous month (*n* = 66418).

Independent variables	Marginal effect				
	Estimated coefficient	Standard error	*p*-value	95% Confidence interval	
Personal characteristics					
Female	−0.885***	0.053	0.000	−0.989	−0.781
Age	0.002	0.001	0.149	−0.001	0.005
Indigenous	−0.115*	0.046	0.012	−0.204	−0.025
Rural	0.021	0.035	0.545	−0.047	0.090
Employed	0.222***	0.047	0.000	0.129	0.314
Cohabiting couples	−0.164***	0.045	0.000	−0.253	−0.075
Education level					
No education	−0.018	0.079	0.820	−0.174	0.138
High school	−0.000	0.041	0.993	−0.081	0.080
University	0.266***	0.072	0.000	0.125	0.406
Regions					
Coast	−0.315***	0.040	0.000	−0.394	−0.236
Amazon	−0.233***	0.034	0.000	−0.300	−0.165
Galapagos	−0.234*	0.105	0.027	−0.440	−0.027
Control variables					
Smoker	0.941***	0.085	0.000	0.776	1.107
Sport	0.105**	0.040	0.010	0.025	0.184

Standard errors are clustered at the household level. ^*^p < 0.05; ^**^p < 0.01; ^***^p < 0.001. Primary education is the reference category for education level. The Andes is the reference category for the region.

### Determinants of alcohol consumption

#### Personal characteristics

The analysis of personal characteristics indicates a significant negative association between being female and alcohol consumption, showing a decrease in the probability of alcohol consumption of 6.6 percentage points (*p*-value: 0.000), suggesting that females are less likely to engage in alcohol consumption compared to their male counterparts. In addition, being employed exhibits a significant positive association of 2.2 percentage points (*p*-value: 0.000), indicating that employment status increases the likelihood of alcohol consumption.

Conversely, the age displays a non-significant association, implying that age, within the observed range, does not significantly impact alcohol consumption. Being indigenous, having a cohabiting couple, and living in a rural area demonstrate non-significant associations, suggesting limited predictive power regarding drinking behavior.

#### Education level

Exploring education levels, individuals with no education and high school education demonstrate a non-significant positive association, while those who have a university education are significantly associated with a higher likelihood of alcohol consumption of 3.0 percentage points (*p*-value: 0.000). All these results are compared with individuals that only have a primary level.

#### Regions

Geographical variations emerge as significant factors influencing alcohol consumption. Residents in the Coast, Amazon, and Galapagos regions display a significant negative association (*p*-value: 0.000), indicating lower likelihoods of alcohol consumption than the Andes region of 2.8, 2.5, and 4.4 percentage points, respectively.

#### Control variables

Examining control variables, being a smoker and practicing a sport exhibit a significant positive association (*p*-value: 0.000) with alcohol consumption of 10.2 and 1.5 percentage points, respectively, suggesting a connection between smoking and increased likelihood of alcohol drinking.

### Determinants of alcohol expenditure

#### Personal characteristics

Being female was linked to a reduction in alcohol expenditure by $0.885 (*p*-value: 0.000). Cohabiting couples exhibited a decrease in spending by $0.164 (*p*-value: 0.000). In addition, being employed was significantly associated with a rise in alcohol expenditure by $0.222 (*p*-value: 0.000). All these results were statistically significant.

In terms of ethnicity, age, and residential location, having an indigenous background, an increase in age by 1 year, and residing in a rural area were not found to significantly impact alcohol expenditure.

#### Education level

Examining education levels, having a university degree was linked to an increase in spending by $0.266 (*p*-value: 0.000). However, individuals who have no education and high school education did not exhibit statistically significant associations with alcohol spending in this model. All these results are compared with individuals that only have a primary level.

#### Regions

Analyzing regional influences, residents on the Coast experienced an associated decrease in spending by $0.315 (*p*-value: 0.000) and living in the Amazon is associated with a decrease by $0.233 *p*-value: 0.000. All results were significant to at least 5% level of significance and compared to Andes region.

#### Control variables

Being a smoker was associated with an increase in spending by $0.941 (*p*-value: 0.000), and engaging in sports led to a rise by $0.105 (*p*-value: 0.010).

## Discussion

Overall, being a smoker and a woman are the variables with the greatest impact on alcohol consumption, although in opposite directions. Smoking is associated with the greatest increase in the probability of consuming alcohol, while being a woman is associated with the greatest reduction in the probability of drinking and spending on alcohol. Consistency is observed in these results, as the probability of buying and spending on alcohol is also higher in men than women (20). In the Ecuadorian context, this gender difference is more noticeable as male heads of households are more likely to spend on alcohol than households led by women (19). Studies from Turkey reinforce these patterns, where male drinkers spend more on alcohol, and cultural expectations lead to lower consumption among women, particularly in rural areas (13, 14). It is also noted that men in Ecuador suffer a higher mortality rate from alcohol-related causes 7.6% than 4% among women, highlighting the need for health policies focused on detection and interventions targeting excessive male drinking (20).

Regarding smoking, the theory suggests there is an interdependence between tobacco and alcohol use. Individuals who drink alcohol are more likely to smoke, and vice versa (20). This relationship is further emphasized in research from Turkey, where concurrent use of alcohol and tobacco is prevalent due to complementary consumption patterns in social settings such as bars and gatherings (12). Similarly, individuals in South Africa exhibit this dual behavior, reinforcing the idea that simultaneous consumption of these substances amplifies spending on both (21). In Ecuador, this behavior persists, as excessive alcohol consumption often coincides with tobacco use in social venues, underscoring the importance of integrated public health strategies to combat both issues simultaneously.

In relation to the level of spending, the results align with the consumption variables. Being a smoker significantly increases expenditures on alcoholic beverages, while being a woman decreases them. In Turkey, men spend approximately 4.23 USD more on alcohol per month than women (20). In Ecuador, the spending gap is also evident, with expenditures decreasing by 0.885 USD for women, reflecting a gender-based difference in spending patterns. These findings underline the importance of tailoring public health policies to address different socioeconomic groups effectively.

The second-largest impact of a socioeconomic determinant is employment status. Having a job is associated with a higher probability of drinking and increased alcohol spending than individuals without employment. These results confirm that financial stability promotes higher alcohol consumption. However, other study suggests that employment can limit participation in drinking due to time constraints, creating a complex dynamic (13). In Ecuador, the effect of employment on alcohol use is similarly nuanced. While employment increases the likelihood of alcohol spending, factors such as living alone or with a partner can influence both consumption and expenditures (22). Cohabiting partners may act as a moderating influence, reducing alcohol consumption within the household.

Education also plays a critical role. In Ecuador, having a university education is associated with a higher probability of alcohol consumption and increased expenditures. However, the impact of education varies across contexts. Studies from Malaysia show that higher education can reduce alcohol consumption, particularly among Chinese ethnic groups, indicating that cultural and economic factors influence these behaviors differently (21, 23). Research found that educated women are more likely to drink, reflecting changing societal norms and economic independence (14). In Ecuador, education often serves as a proxy for income, which explains the positive correlation between education and alcohol consumption: higher incomes enable individuals to spend more on non-essential goods such as alcohol (9).

One of the significant findings is the positive association between sports and alcohol consumption, potentially rooted in Ecuador’s cultural context, where sports participation, particularly in team-based activities, is frequently linked to alcohol use through social interactions, such as post-game celebrations. A systematic review explored a similar association, concluding that sports participation is often correlated with increased alcohol consumption, especially among adolescents. While sports typically promote physical health, they may inadvertently encourage higher alcohol intake within social and team settings, likely due to peer influence, team bonding rituals, and celebratory traditions associated with sports events (24). Although this review focused on a different population, its findings may partially explain the association between sports and alcohol consumption in the Ecuadorian context. Nevertheless, these observations underscore the need for further research to examine the unique dynamics of this relationship in Ecuador as local social contexts may produce distinct patterns in the link between sports and alcohol use.

A key limitation of this study is the availability of updated data in Ecuador. Although the Survey of Living Conditions (ECV) 2014 is somewhat dated, it remains the most recent and comprehensive dataset for analyzing the determinants of alcohol consumption and expenditure. The lack of more recent and detailed data restricted the use of more advanced econometric methods, such as models that require a broader set of predictors to analyze both the decision to consume alcohol and the level of expenditure. Consequently, there is a need for new datasets to monitor trends over time and enable analyses with more comprehensive data.

Another methodological constraint is the exclusion of beer consumption from the analysis. The ECV collects data on beer consumption on a weekly basis, while other alcoholic beverages are measured monthly. This discrepancy in reporting periods complicates the integration of both datasets into a unified framework, potentially overlooking an important component of alcohol consumption patterns in Ecuador. While these limitations may constrain the generalizability of the findings, the study fills an important gap by providing novel insights into alcohol consumption behavior in the Ecuadorian context. Despite the challenges posed by data constraints, the chosen models offer a robust foundation for future research and evidence-based policymaking aimed at addressing alcohol-related issues in the country.

## Implications

This study offers significant theoretical and practical implications for understanding the determinants of alcohol consumption and expenditure in Ecuador. The findings contribute to bridging gaps in the existing literature by providing nuanced insights into socioeconomic factors influencing alcohol-related behaviors in a country where such analyses are limited. The application of robust econometric models, including probit and Tobit, reveals critical determinants such as gender, employment status, education level, and regional disparities. These results enhance the understanding of alcohol consumption in developing economies and offer comparative insights for other Latin American countries with similar socioeconomic contexts.

From a policymaking perspective, the results emphasize the importance of designing targeted interventions to reduce harmful alcohol consumption. Gender-specific strategies could address the higher prevalence of consumption among men while tailoring interventions for women. Additionally, understanding the link between employment and alcohol consumption highlights the need for support mechanisms for employed individuals, who exhibit higher probabilities of consumption. Considering regional disparities, policy interventions should focus on providing greater support for areas with higher rates of alcohol use. Furthermore, the observed interdependence between alcohol and tobacco use underscores the importance of integrated public health strategies that address both issues simultaneously.

By elucidating the socioeconomic factors driving alcohol consumption and expenditure, this study provides a basis for policymakers to design culturally and regionally tailored strategies. Such policies could involve fiscal measures, such as increased taxation on alcoholic beverages, alongside public awareness campaigns and programs directed at the socioeconomic groups most at risk. These interventions would address not only the health consequences but also the broader economic impacts of alcohol consumption, promoting the efficient allocation of resources in Ecuador.

## Conclusion

In conclusion, the design of public policies should prioritize a unified approach to reducing alcohol and tobacco consumption, allowing decision-makers to achieve dual benefits that positively impact both the economic and health sectors (25). Pricing strategies, such as increased taxes on alcoholic beverages, could effectively reduce alcohol consumption and unnecessary spending while reshaping addictive consumer preferences (26).

Given the promising results observed in other regions, pricing policies may represent a key strategy to curb excessive alcohol use in the Americas, including Ecuador. However, further research is necessary to examine the socioeconomic effects of these tax measures, especially in developing countries, to ensure that policies are appropriately targeted and effective in achieving public health objectives. Global efforts to establish “sensible drinking” guidelines, as recommended by organizations such as the National Institute for Health and Care Excellence (NICE) and WHO, highlight the importance of integrated, multi-level policies to mitigate the health and social impacts of excessive alcohol consumption (27).

## Data Availability

The original contributions presented in the study are included in the article/supplementary material, further inquiries can be directed to the corresponding author/s.
